# MicroRNA-224 Suppresses Colorectal Cancer Cell Migration by Targeting Cdc42

**DOI:** 10.1155/2014/617150

**Published:** 2014-04-10

**Authors:** Tao-Wei Ke, Han-Lin Hsu, Yu-Hua Wu, William Tzu-Liang Chen, Ya-Wen Cheng, Chao-Wen Cheng

**Affiliations:** ^1^Institute of Medicine, Chung-Shan Medical University, Taichung 402, Taiwan; ^2^Division of Colorectal Surgery, Department of Surgery, China Medical University Hospital, Taichung 404, Taiwan; ^3^Department of Internal Medicine, Taipei Medical University-Wan Fang Hospital, Taipei 116, Taiwan; ^4^Graduate Institute of Clinical Medicine, College of Medicine, Taipei Medical University, Taipei 110, Taiwan; ^5^PhD Program for Cancer Biology and Drug Discovery, College of Medical Science and Technology, Taipei Medical University, Taipei 110, Taiwan; ^6^Center for Translational Medicine, College of Medical Science and Technology, Taipei Medical University, Taipei 110, Taiwan; ^7^Graduate Institute of Medical Sciences, National Defense Medical Center, Taipei 114, Taiwan

## Abstract

The metastatic spread of tumor cells is the major risk factor affecting the clinical prognosis of colorectal cancer (CRC) patients. The metastatic phenotype can be modulated by dysregulating the synthesis of different structural and functional proteins of tumor cells. Micro(mi)RNAs are noncoding RNAs that recognize their cognate messenger (m)RNA targets by sequence-specific interactions with the 3′ untranslated region and are involved in the multistep process of CRC development. The objective of this study was to investigate the expression and biological roles of miR-224 in CRC. The miR-224 expression level was assessed by a quantitative real-time PCR in 79 CRC and 18 nontumor tissues. Expression levels of miR-224 in CRC tissues were significantly lower than those in nontumor tissues. Its expression level was associated with the mutation status of the APC gene. Ectopic expression of miR-224 suppressed the migratory ability of CRC cell line, but cell proliferation was less affected. Increased miR-224 diminished Cdc42 and SMAD4 expressions at both the protein and mRNA levels and inhibited the formation of actin filaments. Overall, this study indicated a role of miR-224 in negatively regulating CRC cell migration. The expression level of miR-224 may be a useful predictive biomarker for CRC progression.

## 1. Introduction

Colorectal cancer (CRC) is currently one of the leading causes of cancer mortality and the third most common malignant neoplasm worldwide. The major cause of colon cancer-related mortality is due to liver metastasis, and approximately 60% of colon cancer patients are expected to develop metastases [1]. The 5-year survival rate of CRC patients is 91% if the disease is diagnosed while still localized, but only 70% for regional disease, and this declines to 11% once distant metastasis has occurred [2]. In terms of the clinical relevance of a CRC prognosis and the status of metastasis, understanding the underlying mechanisms of CRC tumorigenesis and metastasis will contribute to validating therapeutic targets and improving clinical applications for treating CRC patients.

Micro(mi)RNAs are small RNA molecules (18~25 nucleotides in length) that function as posttranscriptional regulators of gene expression in various species. More than 50% of miRNA genes are located in cancer-associated genomic regions or in fragile sites, indicating that miRNA expression signatures may partly reflect genetic alterations in the progression of human cancers [3]. miRNAs recognize their target(s) by sequence-specific interactions with the 3′ untranslated regions (UTRs) of cognate mRNA targets and repress their translation or modify their stability, which regulates the physiological or disease-associated biological process [4–6]. Several studies reported that miRNAs participate in CRC by modulating certain gene expressions in response to tumorigenesis and metastasis. The tumor-suppressor, Pdcd4, is negatively regulated by miR-21, thereby stimulating invasion, intravasation, and metastasis in CRC [7]. miR-135a and miR-135b are upregulated in CRC, and this was correlated with a reduction in adenomatous polyposis coli (APC) gene expression [8]. In turn, miR-143 and miR-145 were expressed at reduced levels in colon cancer epithelial cells [9], and* in vitro* transfection with either an miR-143 or miR-145 precursor construct led to cell growth inhibition by targeting ERK 5 and insulin receptor substrate-1 [10, 11]. A recent global miRNA expression analysis indicated that miR-143 and miR-145 also appear to function in opposing manners to either inhibit or augment cell proliferation in a metastatic CRC cell model [12]. miR-126, which is also frequently lost in colon cancers, can suppress the growth of neoplastic cells by targeting phosphatidylinositol 3-kinase signaling [13]. Transiently transfected miR-196a can promote significant decreases in adhesion and increases in migration and invasion, but not in proliferation or apoptosis of SW480 colon cancer cells [14].

The dysregulated expression of miR-224 was reported in several human cancers. It was suggested that miR-224 was involved in cell proliferation, migration, invasion, and apoptosis; however, its potential functions as an oncogene or tumor suppressor remain contradictory. The expression level of miRNA-224 is upregulated in hepatocellular carcinoma [15], pancreatic ductal adenocarcinomas [16], breast cancer [17], perineural invasion prostate cancer [18], and clear cell renal cell carcinoma [19], while it is downregulated in breast [20], lung [21], prostate [22], oral [23], and ovarian cancers [24]. In CRC, gain of miR-224 was reported from a global miRNA array analysis [12], and the expression was related to the status of DNA mismatch repair [25], drug resistance [26], and the progression of inflammatory bowel disease [27]. Accordingly, we postulated that miR-224 may play a crucial role in regulating the clinical characteristics of CRC, and its expression level might be a useful predictive biomarker of CRC progression.

## 2. Materials and Methods

### 2.1. Study Subjects

Frozen tissue samples were retrospectively obtained from 79 patients who underwent surgical resection for CRC at the Department of Surgery, Chung Shan Medical University Hospital, between December 2003 and December 2007. Before the resected specimens were collected and used, informed written consent was obtained from all subjects and/or their guardians. The acquisition of samples and their subsequent examination were approved by the institutional review board of Chung Shan Medical University. None of the participants had a previous history of cancer. The clinical stages and pathological features of primary tumors were defined according to the criteria of the American Joint Commission on Cancer. Among these 79 patients, 18 patients with tumor and matched adjacent tissue specimens were firstly applied for the examination of miR-224 expression. Thereafter, 61 patients' specimens with known TP53 and APC status were added and undergone further analysis.

### 2.2. DNA Sequencing

Mutations in the APC and TP53 genes were determined by direct sequencing of polymerase chain reaction (PCR) products amplified from the DNA of tumor samples as previously described [28, 29]. Four sets of oligonucleotide primers (A1: 5′-CAGACTTATTGTGTAGAAGA-3′ and A2: 5′-CTCCTGAAGAAAATTCAACA-3′ for codons 1260 to 1359; B1: 5′-AGGGTTCTAGTTTATCTTCA-3′ and B2: 5′-TCTGCTTGGTGGCATGGTTT-3′ for codons 1339 to 1436; C1: 5′-GGCATTATAAGCCCCAGTGA-3′ and C2: 5′-AAATGGCTCATCGAGGCTCA-3′ for codons 1417 to 1516; and D1: 5′-ACTCCAGATGGATTTTCTTG-3′ and D2: 5′-GGCTGGCTTTTTTGCTTTAC-3′ for codons 1497 to 1596) were used to amplify the mutation cluster region of the APC gene. For the TP53 gene, the primers used in the reactions were E5S (5′-TGCCCTGACTTTCAACTCTG-3′) and E5AS (5′-GCTGCTCACCATCGCTATC-3′) for exon 5, E6S (5′-CTGATTCCTCACTGATTGCT-3′) and E6AS (5′-AGTTGCAAACCAGACCTCAGG-3′) for exon 6, E7S (5′-CCTGTGTTATCTCCTAGGTTG-3′) and E7AS (5′-GCACAGCAGGCCAGTGTGCA-3′) for exon 7, and E8S (5′-GACCTGATTTCCTTACTGCC-3′) and E8AS (5′-TCTCCTCCACCGCTTCTTGT-3′) for exon 8. The PCR products were sequenced using an Applied Biosystems 3100 Avant Genetic Analyzer (Applied Biosystems, Foster City, CA); the PCR primers were also used for sequencing. All APC and TP53 mutations were confirmed by direct sequencing of both DNA strands.

### 2.3. Data Mining

Normalized log2 miR-224 expression data for the nondrug treated NCI-60 tumor cell line collection [30] from CellMiner database (http://discover.nci.nih.gov/cellminer) were analyzed. Among 59 cell lines, 7 CRC cell lines were analyzed with miR-224 expression.

### 2.4. Cell Lines and Culture

Two CRC cell lines were used in this study, including APC-mutated HT-29, CaCO_2_ and SW620 cells, and APC-intact HCT-116 cells. Cells were maintained in RPMI medium supplemented with 10% fetal bovine serum (FBS) and 1% MycoZap antibiotics (Lonza, Switzerland). Cells were cultured at 37°C in a humidified incubator containing 5% CO_2_.

### 2.5. miRNA Transfection

The hsa-miR-224-mimicking sequences were designed and synthesized according to the miRBase database (miR-224 mimics; sense: 5′-CAAGUCACUAGUGGUUCCGUU-3′ and antisense: 5′-CGGAACCACUAGUGACUUGUU-3′) and a scrambled negative control (NC mimics; sense: 5′-UUCUCCGAACGUGUCACGUTT-3′ and antisense: 5′-ACGUGACACGUUCGGAGAATT-3′) (MDBio, Taipei, Taiwan). The miRNA duplexes were resuspended in nuclease-free water at a final concentration of 20 *μ*M and stored at −80°C until used. Cells at 80% confluency were transfected with Lipofectamine 2000 (Invitrogen, Carlsbad, CA) reagent according to the manufacturer's instructions. Six hours after transfection, the medium containing complexes was replaced, and cells were trypsinized and replated for the following studies.

### 2.6. Cell Proliferation Assay

miR-224- or NC mimic-transfected HCT-116 cells were seeded at 2000 cells per well in 96-well plates and incubated overnight. At the indicated time point, 10 *μ*L of 10 mg/mL of the 3-(4,5-dimethylthiazol-2-yl)-2,5-diphenyltetrazolium bromide reagent (Sigma-Aldrich, St. Louis, MO) was added, and the mixture was allowed to react for 3 h at 37°C. Subsequently, the growth medium was removed, and 200 *μ*L of DMSO was added to each well. The absorbance at an optical density (OD) of 450 nm was measured with a spectrophotometer.

### 2.7. siRNA Transfection

The human Cdc42 siRNA (sc-29256, Santa Cruz) was applied for silencing Cdc42 expression according to the manufacturer's instructions. Six hours after transfection, the medium containing complexes was replaced, and cells were trypsinized and replated for the wound-healing and Boyden chamber migration assays. The knockdown efficacy (>70%) had been validated by qRT-PCR and Western blot analysis.

### 2.8. Wound-Healing Assay

A wound-healing assay was performed by seeding 3 × 10^4^ miR-224- or NC mimic-transfected HCT-116 cells into each wound-produced culture insert (400 ± 50 *μ*m wide; Ibidi, Martius, Germany) and incubated overnight. Culture inserts were removed after appropriate cell attachment and washed twice with phosphate-buffered saline (PBS), and complete RPMI medium with 10% FBS was added. Cell migration toward the wounded area was observed and photographed after 24 h. Wound closure (%) was calculated as the area of migrated cells divided by wounded area at 0 h.

### 2.9. Boyden Chamber Migration Assay

In the Boyden chamber migration assays, 1 × 10^5^ cells were seeded in 100 *μ*L serum-free medium atop uncoated membranes with 8 *μ*m pores and translocated toward 500 *μ*L complete media. Cell migration toward the complete media was stained with 4′,6-diamidino-2-phenylindole and photographed after 24 h.

### 2.10. RNA Isolation, Quantitative Reverse-Transcription- (qRT-) PCR

Total RNA was extracted with the TRIzol reagent (Invitrogen) according to the manufacturer's instructions. Total RNA was quantified using a NanoDrop spectrophotometer (Thermo Scientific, Asheville, NC). For the miRNA analysis, 1 *μ*g of RNA was used in a single-round RT reaction with a miScript Reverse Transcription Kit according to protocols provided by the supplier for first-strand complementary (c)DNA synthesis (Qiagen, Valencia, CA). Expression of mature miRNA was detected with a miScript SYBR Green PCR Kit (Qiagen) and presented as −ΔCt (threshold level of fluorescence) values relative to U6-snRNA: ΔCt = Ct (miR-224) − Ct (U6). In tumor samples, the definitions of high and low expression of miR-224 were determined by the mean −ΔCt value (= −5.67) of all patients. Expression levels higher than the mean were defined as high expression, and levels lower than the mean were defined as low expression.

For the messenger (m)RNA analysis, 1 *μ*g of RNA was used in a single-round RT reaction with a GoScript Reverse Transcriptase Synthesis Kit according to the protocols provided by the supplier (Promega, Madison, WI) for first-strand cDNA synthesis. A quantitative real-time PCR was performed using a KAPA SYBR FAST qPCR kit (KAPA Biosystems, Boston, MA) according to the manufacturer's protocol, and results were analyzed using a LightCycler 480 Real-Time PCR System in the Core Facility Center of the Office of Research and Development at Taipei Medical University [31]. The following primers were used: mothers against decapentaplegic homolog 4 (SMAD4): forward 5′-CCATTTCCAATCATCCTGCT-3′ and reverse 5′-ACCTTTGCCTATGTGCAACC-3′; cell division control protein 42 homolog (Cdc42): forward 5′-TACTGCAGGGCAAGAGGATT-3′ and reverse 5′- CCCAACAAGCAAGAAAGGAG-3′; glyceraldehyde 3-phosphate dehydrogenase (GAPDH): forward 5′-TCCACTCACGGCAAATTCAAC-3′ and reverse 5′-TCCACGACATACTCAGCACC-3′; and *β*-actin: forward 5′-CACCATTGGCAATGAGCGGTTC-3′ and reverse 5′-AGGTCTTTGCGGATGTCCACGT-3′ (MDBio). Amplifications were normalized to GAPDH and *β*-actin and calculated using the 2^−ΔΔCt^ method.

### 2.11. Western Blot Analysis

For total cellular protein extraction, CRC cells were washed and then lysed in PRO-PREP protein extraction solution (iNtRON Biotechnology, Kyungki-Do, Korea). Cleared lysates (30 *μ*g/lane) were separated by sodium dodecyl sulfate polyacrylamide gel electrophoresis (SDS-PAGE) on 10x gels and electroblotted onto polyvinylidene difluoride (PVDF) membranes. The membranes were blocked with 0.5% bovine serum albumin (BSA) and then incubated with SMAD4, Cdc42, and GAPDH antibodies. The bound antibodies were detected after incubation with a horseradish peroxidase-conjugated secondary antibody and were visualized by an enhanced chemiluminescence (ECL) system.

### 2.12. Measurement of G-Actin/F-Actin Ratio

To determine the F/G-actin ratio, CRC cells were cultured in 10 cm Petri dishes at a density of 3 × 10^5^ cells and examined with an F/G-actin* in vivo* assay kit (Cytoskeleton, Denver, CO) based on the manufacturer's protocol. Briefly, cells were lysed with cell lysis and F-actin stabilization buffer. After passing through 25G syringes several times, samples were incubated at 37°C for 10 min and then centrifuged at 2000 rpm for 5 min. Subsequently, the supernatant was collected and centrifuged at 10^5^ ×g for 60 min at 37°C. The supernatants (G-actin) were then separated from the pellets (F-actin) and chilled on ice. Pellets were resuspended in the same volume as the supernatants using ice-cold double-distilled (dd)H_2_O containing 1% cytochalasin D and were incubated on ice for 60 min. Equal amounts (15 *μ*L) of samples (supernatant and pellet) were used for the Western blot analysis with an anti-actin antibody.

### 2.13. Statistical Analysis

All data were analyzed using the Statistical Package for the Social Sciences version 19.0 software (SPSS, Chicago, IL). Odds ratios (ORs) and 95% confidence intervals (CIs) of associations of miR-224 expression levels with risk, APC and TP53 gene mutations, and clinicopathological parameters were estimated using multiple logistic regression models. An unpaired Student's *t*-test was used to compare between two groups. A probability of <0.05 was considered statistically significant.

## 3. Results

### 3.1. Downregulation of miR-224 Expression in CRC Specimens and Association with the Mutation Status of the APC Gene

**Figure 1:**
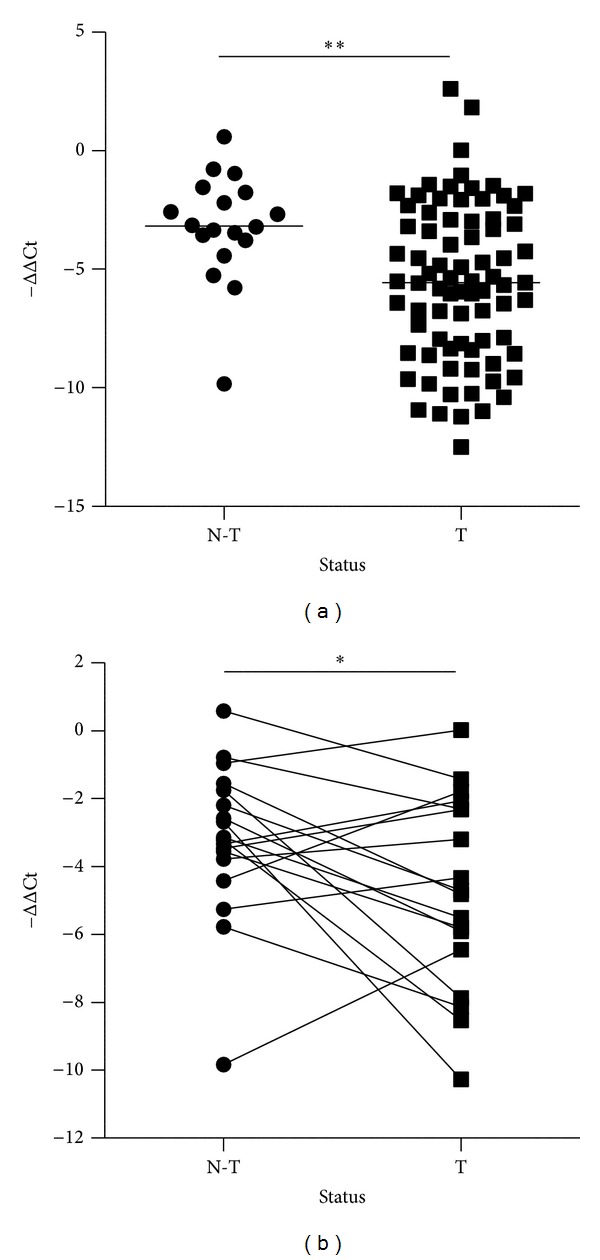
The expression level of miR-224 is repressed in colorectal cancer (CRC) specimens. (a) The level of miR-224 expression in CRC tissues (*n* = 79) was lower than that in control tissues (*n* = 18). (b) The level of miR-224 expression in 18 paired specimens. Values shown (−ΔCt) are related to those of U6-snRNA (**P < 0.01).

In order to validate expression levels of miR-224 in CRC specimens, 97 tissue samples from 79 patients were evaluated by a real-time RT-PCR assay. Results of the statistical analysis of demographic characteristics are presented in Table 1. There were no significant differences in distributions of age and gender between control subjects and CRC patients. The expression level of miR-224 was significantly lower (*P* = 0.0047) in CRC compared to normal tissues (as shown by −ΔCt values of −5.67 ± 3.28 and −3.21 ± 2.28, resp.) (Figure 1(a)). In comparison with 18 paired specimens, the expression level of miR-224 was also lower (*P* = 0.0447) in the tumor samples (−ΔCt values of tumor and adjacent samples; −4.75 ± 2.83 and −3.21 ± 2.28, resp.) (Figure 1(b)).

The total primary CRC samples were divided into two groups using the mean value of miR-224 (mean value: −ΔCt = −5.67; miR-224-high and -low, resp.), and distributions of the age, gender, clinical stages, tumor T status, lymph node, and distant metastasis and APC and TP53 gene mutation status in CRC samples are given in Table 2. A significantly larger number of CRC patients carrying the mutated APC gene were found in the miR-224-high group compared to the miR-224-low group (OR = 3.643; 95% CI: 1.297~10.228, *P* = 0.015). However, no associations were found for the miR-224-high and -low groups with age, gender, clinical stages, tumor T status, lymph node, and distant metastasis or the TP53 gene mutation status.

### 3.2. A Higher miR-224 Expression Level Suppressed the Migratory Ability of CRC Cell Line

**Figure 2:**
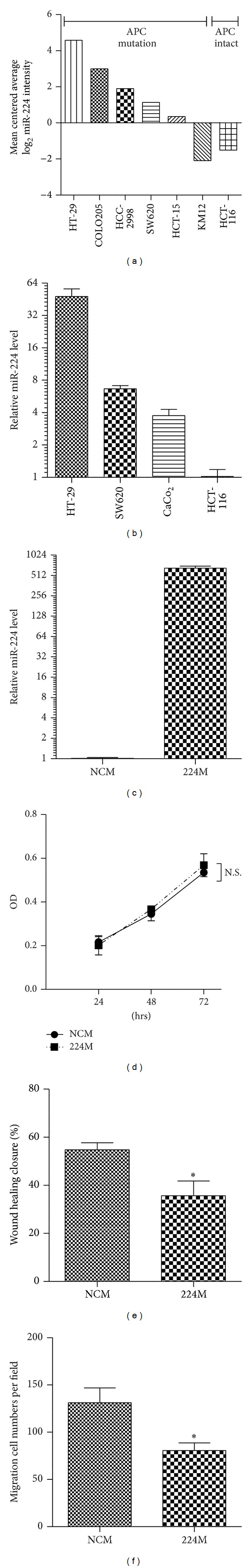
Increased miR-224 expression suppressed the colorectal cancer (CRC) cell migration ability but not cell proliferation. (a) Normalized log_2_ miR-224 expression data in CRC cell lines from the nondrug treated NCI-60 tumor cell line collection. (b) Real-time RT-PCR analysis of miR-224 expression levels in HT-29 and HCT-116 cells. (c) Real-time RT-PCR analysis of miR-224 expression levels in NCM- and 224 M-transfected HCT-116 cells. (d) Proliferation potential of NCM- and 224 M-transfected HCT-116 cells. (e, f) Migratory ability of NCM- and 224 M-transfected HCT-116 cells (*P < 0.05; **P < 0.01).

The expression profiles of miR-224 were analyzed in nondrug treated NCI-60 tumor cell line CellMiner Database. All 7 CRC cell lines in NCI-60 panel were selected and analyzed; APC-mutated CRC cell lines, except for KM12, tend to express more miR-224 than APC gene-intact HCT-116 cell line (Figure 2(a)). In addition, four human malignant CRC cell lines with different statuses of the APC gene were selected to validate the miR-224 expression levels. Results also showed that the APC gene-mutated HT-29, CaCO_2_, and SW620 cell lines presented higher miR-224 expression level than the APC gene-intact HCT-116 cell line (Figure 2(b)). To gain further insights into the role of miR-224 in CRC malignancy, we transfected either NC or miR-224 mimics into HCT-116 cells to analyze the biological effects on cell growth and the migratory ability. HCT-116 cells transfected with miR-224 mimics expressed an approximately 600-fold higher miR-224 level than NC mimics at 48 h after transfection (Figure 2(c)). The cell growth rate showed no significant differences in HCT-116 cells transfected with either the NC or miR-224 mimic (Figure 2(d)). Compared to NC mimic-transfected HCT-116 cells, miR-224 mimic-transfected cells exhibited a significant reduction in the migration ability in the* in vitro* wound-closure assay and Boyden chamber migration assay (Figures 2(e) and 2(f)). It should be noted that HT-29 cells showed a very low migration ability in the wound healing assay (<5% wound healing closure at 24 h, data not shown), which may be partly caused by the APC mutation mediated miR-224 overexpression. These results suggest that the increased miR-224 expression negatively regulates the migratory ability of CRC cells.

### 3.3. Ectopic Expression of miR-224 Diminished Cdc42 and SMAD4 Expressions and Inhibited the Formation of Actin Filaments

**Figure 3:**
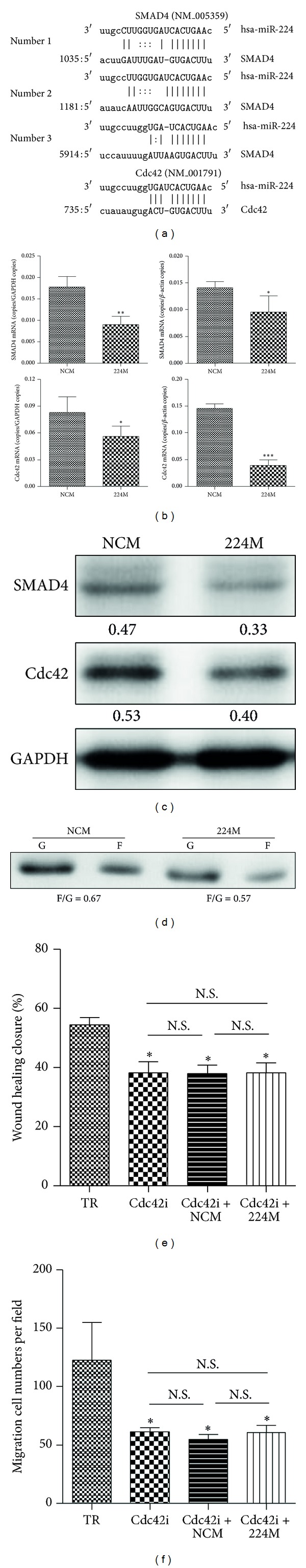
Increased miR-224 expression diminished Cdc42 and SMAD4 expressions and inhibited the formation of actin filaments. (a) Prediction of miR-224 targeting mRNA sequences on SMAD4 and Cdc42 in the Sloan-Kettering Cancer Center Human MicroRNA Targets Database. (b, c) mRNA and protein levels of SMAD4 and Cdc42 in NCM- and 224 M-transfected HCT-116 cells (*P < 0.05; **P < 0.01). (d) Representative Western blot of G- and F-actin from NCM- and 224 M-transfected HCT-116 cells. Data are from one of three experiments. (e, f) Migratory ability of Cdc42 silenced HCT-116 cells transfected with NCM and 224 M (*P < 0.05, compared to TR alone).

Because of the miR-224 participated in regulating CRC migration ability, its putative targets in CRC may be involved in metastatic progression. SMAD4 and Cdc42 are both putative miR-224 targets, which were previously reported to be related to CRC metastasis. In searching through the Sloan-Kettering Cancer Center Human MicroRNA Targets Database [32], miR-224 was predicted with good mirSVR scores when targeting three different sequence sites on SMAD4 and one on Cdc42 (Figure 3(a)). Therefore, we further analyzed whether ectopic expression of miR-224 affected endogenous expressions of SMAD4 and Cdc42. Forty-eight hours after transfection, both the mRNA and protein levels of SMAD4 and Cdc42 were noted to be suppressed in miR-224-transfected HCT-116 cells (Figures 3(b) and 3(c)). The phenomenon of a cancer cell migrating through modifying the shape and stiffness of the surrounding tissue structures is controlled by dynamic changes in the actin cytoskeleton and leads to interactions with extracellular matrix (ECM) substrates. Cdc42 is a small GTP-binding protein which can regulate the formation of actin filament-based structures. As shown in Figure 3(d), miR-224 mimic-transfected HCT-116 cells showed decreased formation of filamentous actin compared to NC mimics (Figure 3(d)). In addition, silencing Cdc42 had decreased the migration of HCT-116 as compared to transfection reagent (TR) alone. Cotransfected with 224 M in Cdc42i, treated cells showed no significant differences as compared to those cotransfected either with NCM or Cdc42i alone (Figures 3(e) and 3(f)). These findings suggest that miR-224 may regulate CRC cell migration, at least in part, through inhibiting Cdc42 expression and suppressing filamentous actin-mediated cell migration.

## 4. Discussion

In the current study, according to a quantitative real-time PCR analysis, the expression level of miR-224 was lower in CRC specimens compared to normal tissues. The miR-224 expression level was not associated with age, gender, clinical parameters, or the TP53 status, while the expression level was associated with the mutation status of the APC gene. The HCT-116 cell migration ability was suppressed by ectopic expression of miR-224 through diminishing Cdc42-mediated filamentous actin formation. These findings suggest that downregulation of miR-224 increased cancer cell migration in CRC and thus may, at least in part, participate in CRC metastasis.

The expression level of miR-224 increased between normal tissues and the early stage of CRC but did not show significant differential expression between the early and late stages in a global CRC specimen miRNA analysis [12]. Recently, Yuan et al. found that levels of miR-224 were reduced in metastatic, compared to nonmetastatic, CRC cells. Expression levels in human tumor samples were inversely correlated with the tumor stage, metastasis to lymph nodes, and patient survival times, but no differences were observed between normal and tumor samples [33]. In this study, miR-224 exhibited lower expression in tumor samples than normal samples (Figure 1). In addition, its expression level was not related to clinical parameters (i.e., age, gender, TMN stage, or differentiation) or the status of the TP53 mutation, while it was related to the status of the APC mutation (Table 2). These findings suggest that differences in miR-224 expression levels in the pathogenesis of CRC may be related to racial and environmental diversity; however, this inference requires further investigation.

Regulation of miR-224 expression was reported in several different cell types. In transforming growth factor (TGF)-*β*1-stimulated mouse ovarian granulosa cells, miR-224 expression was regulated by the TGF-*β*/SMADs pathway [34]. Overexpression of Src in mouse embryonic Cx43Ko brain cells also increased miR-224 expression [35]. In addition, the E2-conjugating enzyme, Ubc9, that transfers an activated small ubiquitin-related modifier to protein substrates can suppress miR-224 expression in breast cancer cells. In the current study, a significantly higher number of CRC patients carrying the mutated APC gene were found in the miR-224-high group compared to the miR-224-low group. In addition, miR-224 was also more highly expressed in three APC-mutated cell lines than APC-intact HCT-116 cells (Figure 2(a)). APC is a negative regulator of the Wnt/ß-catenin/TCF pathway, which is involved in both carcinogenesis [36] and normal intestinal homeostasis [37]. Mutated APC can cause familial adenomatous polyposis and is mutated in the vast majority of all sporadic colorectal cancers [38]. In the absence of Wnt signaling, *β*-catenin undergoes ubiquitin-mediated degradation by a multiprotein destruction complex, composed of the tumor suppressor APC, Axin, protein phosphatase 2A, glycogen synthase kinase 3, and casein kinase 1*α* [39]. Defect of APC leads Wnt signaling constitutive transcriptional activation through the nuclei accumulation of a *β*-catenin/TCF transcription factor complex [40]. The genetic location of miRNA-224 is within a 200 kb region of Xq28 in close proximity to miR-452 within the GABRE gene and is flanked by the MAGEA4 and MAGEA5 cancer antigens [34]. In addition, the GABRE gene had been reported exclusively overexpressed with Wnt pathway activation [41]. These evidences suggested that the increase of miR-224 in APC-mutated CRC cells may be regulated by the constitutive activation of Wnt/ß-catenin/TCF pathway. Therefore, the APC mutation activates the Wnt signal pathway and mediates an increase in miR-224, which may serve as a negative regulator of cell motility and metastasis.

The multifaceted involvement, including cell proliferation, migration, invasion, and apoptosis, of miR-224 in malignancy was reported. It was reported that miR-224 was the most significantly upregulated miRNA in HCC patients, and it was revealed that miR-224 increased both apoptotic cell death and proliferation [42]. Here, ectopic miR-224 expression decreased HCT-116 cell migration but not cell proliferation (Figures 2(c)–2(f)); this indicates that expression of miR-224 in CRC manifests the potential for the cell migration ability. However, there was no significant decrease in the miR-224 level observed between metastatic and nonmetastatic patients in this study. In addition to the limited number of metastatic patients, only primary tumor samples were collected to evaluate miR-224 in this study, thus limiting our current findings on the clinical association between miR-224 expression levels and metastasis [29].

Several putative targets of miR-224 were validated to be involved in regulating cell proliferation, migration, and invasion, such as apoptosis inhibitor-5 [42], RKIP [17], SMAD4 [34], Cdc42, and CXCR4 [43]. In this study, miR-224 showed participation in CRC cell migration; its putative targets related to the cell-migration ability, SMAD4 and Cdc42, were selected for further investigation. Ectopic expression of miR-224 inhibited both the mRNA and protein expressions of SMAD4 and Cdc42 (Figures 3(b) and 3(c)), which may result in suppression of the* in vitro* HCT-116 cell migration ability (Figures 2(e) and 2(f)). It was reported that loss of SMAD4 in CRC cells plays an important role in increasing the tumorigenic and metastatic potential [44]. A recent study indicated that miR-224 can inhibit SMAD4 expression to increase SW480 cell migration and invasion ability [45]. However, loss in the TGF-*β* receptor or SMAD4 commonly occurs in sporadic CRC patients and cell lines, such as SW480, HT-29, SW620 (absent SMAD4 protein and allelic loss at SMAD4), CaCO_2_ (SMAD4 point mutations and rapid SMAD4 protein degradation), and HCT-116 (mutation in TGFBIIR) [46]. Therefore, miR-224 diminishing the HCT-116 cell-migration ability might not be mediated through direct targeting of the TGF-*β*/SMAD4 pathway. In contrast, Cdc42 is involved in regulating the rearrangement of the actin cytoskeleton, and it was found to be overexpressed with a high incidence in CRC samples [47]. Overexpression of miR-224 reduced cytoskeletal composition changes from global- to filamentous-actin (Figure 3(d)), which suppressed the cell motility and the formation of lamellipodia, thereby diminishing CRC migration. In addition to directly targeting Cdc42, decreased levels of miR-224 also increased methyl-CpG-binding domain protein 2 gene expression, thereby suppressing maspin and promoting CRC growth and metastasis [33].

In summary, we investigated expression levels of miR-224 in CRC clinical specimens and its biological functions. The expression levels of miR-224 were lower in CRC tissues and its expression level was associated with the mutation status of the APC gene. A reduction in mR-224 expression may cause epigenetic regulation of CRC metastasis through targeting Cdc42 and may be considered as a potential predictive biomarker for CRC progression.

## Figures and Tables

**Table 1:** Distributions of demographic characteristics of the 18 controls and 79 patients with colorectal cancer.

Variable	Controls (*n* = 18)	Patients (*n* = 79)	*P* value
Age (Mean ± SD)	58.72 ± 16.01	65.42 ± 15.07	0.0958

	*n* (%)	*n* (%)	

Gender			
Male	8 (44.4%)	35 (44.3%)	0.9914
Female	10 (56.6%)	44 (55.7%)	
Stage			
I		9 (13.8%)	
II		34 (40.5%)	
III		30 (36.2%)	
IV		6 (9.5%)	
Tumor T status			
≤2		11 (13.9%)	
>2		68 (86.1%)	
Lymph node status			
N0		44 (55.7%)	
N1 + N2		35 (44.3%)	
Metastasis			
M0		73 (92.4%)	
M1		6 (7.6%)	
APC			
Normal		55 (69.6%)	
Mutated		24 (30.4%)	
TP53			
Normal		22 (27.8%)	
Mutated		57 (72.2%)	

**Table 2:** Clinical and genetic status and miR-224 expression level frequency in 79 colorectal cancer (CRC) patients.

Variable	MiR-224-low^a^ (*n* = 40)	MiR-224-high^b^ (*n* = 39)	Odds ratio (95% CI)	*P* value
Age				
≤68 (median age)	19 (47.5%)	21 (53.8%)	1.29 (0.533~3.121)	0.573
>68 (median age)	21 (52.5%)	18 (46.2%)		
Gender				
Female	21 (52.5%)	14 (35.9%)	0.507 (0.206~1.248)	0.140
Male	19 (47.5%)	25 (64.1%)		
Clinical stage				
Stage I/II	23 (57.5%)	20 (51.3%)	1.285 (0.529~3.121)	0.579
Stage III/IV	17 (42.5%)	19 (48.7%)		
Tumor T status				
≤2	5 (12.5%)	6 (15.4%)	0.786 (0.219~2.822)	0.712
>2	35 (87.5%)	33 (84.6%)		
Lymph node metastasis				
No	24 (60%)	20 (51.3%)	1.425 (0.584~3.475)	0.436
Yes	16 (40%)	19 (48.7%)		
Distant metastasis				
No	36 (90%)	37 (94.9%)	0.487 (0.084~2.823)	0.422
Yes	4 (10%)	2 (5.1%)		
APC				
Normal	33 (82.5%)	22 (56.4%)	3.643 (1.297~10.228)	**0.015**
Mutated	7 (17.5%)	17 (43.6%)		
TP53				
Normal	11 (27.5%)	11 (28.2%)	0.966 (0.361~2.583)	0.945
Mutated	29 (72.5%)	28 (71.8%)		

^a^Samples expressed miR-224 at lower than the average −ΔCt value.

^b^Samples expressed miR-224 at higher than the average −ΔCt value.

CI: confidence interval.

## References

[1] Stangl R, Altendorf-Hofmann A, Charnley RM, Scheele J (1994). Factors influencing the natural history of colorectal liver metastases. *The Lancet*.

[2] Jemal A, Siegel R, Xu J, Ward E (2010). Cancer statistics, 2010. *CA Cancer Journal for Clinicians*.

[3] Zhang B, Pan X, Cobb GP, Anderson TA (2007). microRNAs as oncogenes and tumor suppressors. *Developmental Biology*.

[4] Zamore PD, Haley B (2005). Ribo-gnome: the big world of small RNAs. *Science*.

[5] Bartel DP (2009). MicroRNAs: target recognition and regulatory functions. *Cell*.

[6] Sun G, Li H, Rossi JJ (2007). Cloning and Detecting Signature MicroRNAs from Mammalian Cells. *Methods in Enzymology*.

[7] Asangani IA, Rasheed SAK, Nikolova DA (2008). MicroRNA-21 (miR-21) post-transcriptionally downregulates tumor suppressor Pdcd4 and stimulates invasion, intravasation and metastasis in colorectal cancer. *Oncogene*.

[8] Nagel R, le Sage C, Diosdado B (2008). Regulation of the adenomatous polyposis coli gene by the miR-135 family in colorectal cancer. *Cancer Research*.

[9] Michael MZ, O’Connor SM, van Holst Pellekaan NG, Young GP, James RJ (2003). Reduced accumulation of specific microRNAs in colorectal neoplasia. *Molecular Cancer Research*.

[10] Akao Y, Nakagawa Y, Naoe T (2006). MicroRNAs 143 and 145 are possible common onco-microRNAs in human cancers. *Oncology Reports*.

[11] Shi B, Sepp-Lorenzino L, Prisco M, Linsley P, Deangelis T, Baserga R (2007). Micro RNA 145 targets the insulin receptor substrate-1 and inhibits the growth of colon cancer cells. *Journal of Biological Chemistry*.

[12] Arndt GM, Dossey L, Cullen LM (2009). Characterization of global microRNA expression reveals oncogenic potential of miR-145 in metastatic colorectal cancer. *BMC Cancer*.

[13] Guo C, Sah JF, Beard L, Willson JKV, Markowitz SD, Guda K (2008). The noncoding RNA, miR-126, suppresses the growth of neoplastic cells by targeting phosphatidylinositol 3-kinase signaling and is frequently lost in colon cancers. *Genes Chromosomes and Cancer*.

[14] Schimanski CC, Frerichs K, Rahman F (2009). High miR-196a levels promote the oncogenic phenotype of colorectal cancer cells. *World Journal of Gastroenterology*.

[15] Li Q, Wang G, Shan J-L (2010). MicroRNA-224 is upregulated in HepG2 cells and involved in cellular migration and invasion. *Journal of Gastroenterology and Hepatology*.

[16] Mees ST, Mardin WA, Sielker S (2009). Involvement of CD40 targeting miR-224 and miR-486 on the progression of pancreatic ductal adenocarcinomas. *Annals of Surgical Oncology*.

[17] Huang L, Dai T, Lin X (2012). MicroRNA-224 targets RKIP to control cell invasion and expression of metastasis genes in human breast cancer cells. *Biochemical and Biophysical Research Communications*.

[18] Prueitt RL, Yi M, Hudson RS (2008). Expression of microRNAs and protein-coding genes associated with perineural invasion in prostate cancer. *Prostate*.

[19] Liu H, Brannon AR, Reddy AR (2010). Identifying mRNA targets of microRNA dysregulated in cancer: with application to clear cell Renal Cell Carcinoma. *BMC Systems Biology*.

[20] Giricz O, Reynolds PA, Ramnauth A (2012). Hsa-miR-375 is differentially expressed during breast lobular neoplasia and promotes loss of mammary acinar polarity. *Journal of Pathology*.

[21] Yanaihara N, Caplen N, Bowman E (2006). Unique microRNA molecular profiles in lung cancer diagnosis and prognosis. *Cancer Cell*.

[22] Mavridis K, Stravodimos K, Scorilas A (2013). Downregulation and prognostic performance of microRNA 224 expression in prostate cancer. *Clinical Chemistry*.

[23] Scapoli L, Palmieri A, lo Muzio L (2010). MicroRNA expression profiling of oral carcinoma identifies new markers of tumor progression. *International Journal of Immunopathology and Pharmacology*.

[24] Iorio MV, Visone R, di Leva G (2007). MicroRNA signatures in human ovarian cancer. *Cancer Research*.

[25] Oberg AL, French AJ, Sarver AL (2011). MiRNA expression in colon polyps provides evidence for a Multihit model of colon cancer. *PLoS ONE*.

[26] Mencia N, Selga E, Noé V, Ciudad CJ (2011). Underexpression of miR-224 in methotrexate resistant human colon cancer cells. *Biochemical Pharmacology*.

[27] Olaru AV, Yamanaka S, Vazquez C (2013). MicroRNA-224 negatively regulates p21 expression during late neoplastic progression in inflammatory bowel disease. *Inflammatory Bowel Diseases*.

[28] Chen TH, Huang CC, Yeh KT (2012). Human papilloma virus 16 E6 oncoprotein associated with p53 inactivation in colorectal cancer. *World Journal of Gastroenterology*.

[29] Chen TH, Chang SW, Huang CC (2013). The prognostic significance of APC gene mutation and miR-21 expression in advanced stage colorectal cancer. *Colorectal Disease*.

[30] Reinhold WC, Sunshine M, Liu H (2012). CellMiner: a web-based suite of genomic and pharmacologic tools to explore transcript and drug patterns in the NCI-60 cell line set. *Cancer Research*.

[31] Lin JA, Fang SU, Su CL (2014). Silencing glucose-regulated protein 78 induced renal cell carcinoma cell line G1 cell-cycle arrest and resistance to conventional chemotherapy. *Urologic Oncology*.

[32] John B, Sander C, Marks DS (2006). Prediction of human microRNA targets. *Methods in Molecular Biology*.

[33] Yuan K, Xie K, Fox J (2013). Decreased levels of miR-224 and the passenger strand of miR-221 increase MBD2, suppressing maspin and promoting tumor growth and metastasis in mice. *Gastroenterology*.

[34] Yao G, Yin M, Lian J (2010). MicroRNA-224 is involved in transforming growth factor-*β*-mediated mouse granulosa cell proliferation and granulosa cell function by targeting Smad4. *Molecular Endocrinology*.

[35] Li X, Shen Y, Ichikawa H, Antes T, Goldberg GS (2009). Regulation of miRNA expression by Src and contact normalization: effects on nonanchored cell growth and migration. *Oncogene*.

[36] Scholer-Dahirel A, Schlabach MR, Loo A (2011). Maintenance of adenomatous polyposis coli (APC)-mutant colorectal cancer is dependent on Wnt/*β*-catenin signaling. *Proceedings of the National Academy of Sciences of the United States of America*.

[37] Fevr T, Robine S, Louvard D, Huelsken J (2007). Wnt/*β*-catenin is essential for intestinal homeostasis and maintenance of intestinal stem cells. *Molecular and Cellular Biology*.

[38] Lin J, Wang X, Dorsky RI (2011). Progenitor expansion in apc mutants is mediated by Jak/Stat signaling. *BMC Developmental Biology*.

[39] McCartney BM, Näthke IS (2008). Cell regulation by the Apc protein. Apc as master regulator of epithelia. *Current Opinion in Cell Biology*.

[40] Korinek V, Barker N, Morin PJ (1997). Constitutive transcriptional activation by a *β*-catenin-Tcf complex in APC(-/-) colon carcinoma. *Science*.

[41] Gokhale A, Kunder R, Goel A (2010). Distinctive microRNA signature of medulloblastomas associated with the WNT signaling pathway. *Journal of Cancer Research and Therapeutics*.

[42] Wang Y, Lee ATC, Ma JZI (2008). Profiling microRNA expression in hepatocellular carcinoma reveals microRNA-224 up-regulation and apoptosis inhibitor-5 as a microRNA-224-specific target. *Journal of Biological Chemistry*.

[43] Zhu S, Sachdeva M, Wu F, Lu Z, Mo Y-Y (2010). Ubc9 promotes breast cell invasion and metastasis in a sumoylation- independent manner. *Oncogene*.

[44] Zhang B, Halder SK, Kashikar ND (2010). Antimetastatic role of smad4 signaling in colorectal cancer. *Gastroenterology*.

[45] Zhang GJ, Zhou H, Xiao HX, Li Y, Zhou T (2013). Up-regulation of miR-224 promotes cancer cell proliferation and invasion and predicts relapse of colorectal cancer. *Cancer Cell International*.

[46] Woodford-Richens KL, Rowan AJ, Gorman P (2001). SMAD4 mutations in colorectal cancer probably occur before chromosomal instability, but after divergence of the microsatellite instability pathway. *Proceedings of the National Academy of Sciences of the United States of America*.

[47] Gómez del Pulgar T, Valdés-Mora F, Bandrés E (2008). Cdc42 highly expressed in colorectal adecarcinoma and downregulates ID4 through an epigenetic mechanism. *International Journal of Oncology*.

